# Practical Uncertainty Limits to the Mass Determination of a Piston-Gage Weight

**DOI:** 10.6028/jres.093.149

**Published:** 1988-08-01

**Authors:** R. S. Davis, B. E. Welch

**Affiliations:** National Bureau of Standards Gaithersburg, MD 20899

**Keywords:** analytical balance, calibration, mass, piston-gage, surface effects, weighing, weighing errors

## Abstract

The mass of a 590-g piston-gage weight was determined with a standard error of 0.057 mg (0.1 ppm). The sources of error are carefully examined. These include air-buoyancy corrections, physically adsorbed surface moisture, and air-convection within the weighing chamber. We conclude that significant improvement cannot be realized with the conventional weighing techniques available to most piston-gage users.

## 1. Introduction

The equilibrium pressure in a piston gage is related in a fundamental way to the gravitational force on the rotating parts and area of the piston. The gravitational force, in turn, is determined by the product of the local gravitational acceleration and the sum of the masses of each rotating component. When piston gages are operated in the absolute mode, additional forces associated with the atmosphere, such as air buoyancy, are absent. These forces must be considered in gage-mode operation.

In this paper, we will focus on the problem of assigning mass values to piston-gage weights of about 590-g nominal mass. The goal of these measurements is that the uncertainty in the mass calibration lead to an error in the maximum pressure generated by the rotating assembly of less than 1 ppm (1 × 10^−6^). This level of accuracy is motivated by research underway at NBS which seeks to achieve unprecedented levels of accuracy in the calibration of selected piston gages. The standard used in these measurements is a mercury manometer which has an error of the order of 1 ppm in the pressure range from 10 to 130 kPa [1].

Most of the rotating mass of a piston gage is in a stack of 590-g weights. The uncertainty of the mass of such a stack of weights is the direct sum of uncertainties systematic to the calibration of each weight, combined with the root-sum-square of the random uncertainty associated with each weight. Thus to achieve the desired total uncertainty, care must be taken in characterizing both types of error. We describe in detail how we have evaluated the total uncertainty of our mass measurements. Particular attention will be paid to errors which are systematic to a given measurement technique and thus difficult to detect. These errors can amount to 1 ppm and are, therefore, important to the end result. By contrast, the NBS mass calibration service typically provides an uncertainty of 0.04 ppm (one standard deviation) for calibrations of a single 500-g standard of the highest commercially-available quality.

## 2. Assignment of Mass (Ideal)

A mass *m*_X_ is assigned to a piston-gage weight by comparison with a known standard of mass *m*_S_. The comparison is done under ambient atmospheric conditions. The following formula is then used to determine *m*_X_ [2]:

$$m_{X}=m_{S}\cdot (1-\rho /\rho_{S})/(1-\rho /\rho_{X})+\Delta ,$$ (1)

where

*ρ* = density of the air within the balance case (≈1.2 mg/cm^3^),
*ρ*_S_ = density of the standard (≈8 g/cm^3^),
*ρ*_X_ = density of the piston-gage weight (≈7.8 g/cm^3^),
Δ = difference between balance readings corresponding to S and X (− 2 mg < Δ < 2 mg).

We can estimate the expected uncertainty in *m*_X_ by simple propagation of error through eq (1). The standard S is calibrated in SI units of mass with an uncertainty of about 0.04 ppm. (In this paper, we will use the BIPM recommendation for combining errors. All uncertainties are given as estimates of one standard deviation.) This uncertainty propagates directly as a 0.04 ppm uncertainty in *m*_X_. The air density *ρ* becomes increasingly important as the density of X diverges from that of S. In the present situation, the two densities are nearly equal. Therefore, a measurement of *ρ* to a modest 1 percent will propagate as an error of less than 0.04 ppm in *m*_X_. The air density *ρ* is determined from measurements of barometric pressure, temperature within the balance case, and relative humidity. With these data, *ρ* can be determined to sufficient accuracy through the use of an equation of state for moist air [3]. The balance used has a precision of 0.040 mg (one standard deviation). By averaging several repeated measurements, the contribution of error in Δ to the final result can be made arbitrarily small, *so long as all errors are randomly distributed.* Most of this article will be devoted to examining the sources and magnitudes of all known errors in Δ.

The contribution of uncertainty in *ρ*_S_ and *ρ*_X_ to the total error in *m*_X_ deserves discussion. Suppose a weight T is calibrated in air of density *ρ*_1_. Since the calibration is done in air, the mass value *m*_T_ assigned to T will depend on the density $\rho_{T}^{\prime }$ which is assigned to the weight. Now suppose that the actual density of T is *ρ*_T_; whenever T is used as a standard in subsequent mass or force measurements, use of the assigned mass value *m*_T_ and the assumed density $\rho_{T}^{\prime }$ will produce an error whose magnitude is

$$E=|(\rho_{1}-\rho_{2})(1/\rho_{T}^{\prime }-1/\rho_{T})|,$$ (2)

where *ρ*_2_ is the air density during the subsequent measurement. From eq (2) it is clear that the error *E* will be small if *ρ*_1_ ≈ *ρ*_2_. This is always true for the standard S, which is always used at normal laboratory conditions. Thus the density of S need only be known to an uncertainty of about 1 percent and a value of density obtained from a metals handbook will suffice. The piston-gage weight X, however, will sometimes be used in vacuum (*ρ*_2_ ≈ 0). In order that *E* be less than 0.05 ppm when the piston-gage is operated in the absolute mode, *ρ*_X_ must be uncertain to no more than 0.03 percent. To achieve the needed accuracy, the densities of the piston-gage weights were measured by hydrostatic weighing.

## 3. Known Problems

When using a conventional commercial balance, eq (1) may be considered an accurate mathematical model to a level of about 0.5 ppm (0.3 mg for a 590-g weight). In particular, errors in Δ can usually be considered random at this level. Averaging of repeated measurements of Δ may produce a more accurate value below 0.5 ppm, but this assumption must be tested. The problems indicated in sections 3.1–3.5 may occur.

### 3.1 Between-Times Error

Identical series of measurements taken on different days may differ by significantly more than the combined random uncertainties of each day. The additional uncertainty may itself be randomly distributed, however. If this is so, it is referred to as a “between-times” error (with a characteristic standard deviation *σ*_b_) to distinguish it from the random error observed within a single series of measurements (with a characteristic standard deviation *σ*_w_) [4]. The presence of a significant between-times component of error is usually unforeseen and its source impossible to determine. The total random error *σ*_t_ in the average of a series of duplicated measurements will be:

$${\left(\frac{\sigma_{b}^{2}}{m}+\frac{\sigma_{w}^{2}}{nm}\right)}^{1/2},$$

where *n* is the number of repeated measurements within each run and *m* is the number of runs on separate days. If only one day’s measurements are used, then *m* = 1. In this case, it is clear that by increasing *n* one will arrive at a point where the total error is dominated by *σ*_b_. At this point, increasing *n* further is wasted effort. It is usual to assume that *σ*_b_ is negligible. This assumption should be checked, especially when using a new measurement system or when relying on statistical averaging to reduce uncertainties by a factor of three or more.

### 3.2 Surface Moisture

The surface of stainless steel in air is covered with moisture. At room temperature, the mass per unit area of this moisture layer will depend on the relative humidity and the surface finish of the alloy. Kochsiek has found that metals generally have 0.1 to 0.3 *μ*g/cm^2^ of adsorbed moisture at 50 percent relative humidity and at room temperature [5]. His studies were done gravimetrically. Yoshimori et al. used chemical analysis to determine the moisture given up by metals into a pure argon atmosphere as a function of temperature [6]. The lowest temperature studied was 100 °C. Thus their results represent an upper limit to the moisture which would be given up at room temperature. This being the case, their results are consistent with those of Kochsiek. Thus weights calibrated at laboratory conditions and which have surface area and finish different from the standard will have a mass which, at some level, will depend on the ambient relative humidity. The magnitude of this effect is difficult to calculate quantitatively but should appear as a correlation of the calibration results with relative humidity.

When the calibrated weight is then used in a vacuum (relative humidity of zero) its mass will be lower to the extent that adsorbed moisture is removed. Based on [4] and [5], this effect may be negligible or it may be as great as 0.2 ppm in the mass of a piston-gage weight. A definitive answer to this question is beyond the scope of the present study.

### 3.3 Weight Stability

The question of surface moisture can be thought of as one aspect of weight stability. The best-quality commercial weights have physical characteristics which conform to ASTM Type 1 Grade S [7]. A weight with no sharp edges and made of a single-piece of non-magnetic stainless steel conforms to these specifications so long as its surface has the following properties: 1. Area does not exceed twice the area of a cylinder of equal height and diameter; 2. Highly polished except for markings and adjustment area, free of pits and pores. According to the first surface requirement, the surface area of a 590-g weight may not exceed 200 cm^2^. As can be seen in table 1, the piston-gage weight fails to meet this specification. As for the second requirement, our piston-gage weights are free from pits and pores but are not polished. Thus the piston-gage weights may not be as stable as the best quality laboratory weights.

### 3.4 Gravitational Gradient

Weighing with an analytical balance relies on the existence of a local, constant, gravitational acceleration. The actual value of this acceleration cancels out of eq (1). Cancellation will not be perfect, however, if the centers of mass of the standard and unknown are placed on the balance at different elevations with respect to the earth. The lack of cancellation is due to the gravitational gradient at the earth’s surface (about −0.003 ppm/cm [8]). If left uncorrected, the gravitational gradient would produce only a small systematic error.

### 3.5 Lack of Thermal Equilibrium

Equation (1) assumes that both the standard and unknown are in thermal equilibrium with the air in the balance chamber. Violation of this condition may produce non-negligible systematic errors in the results.

These errors are presumably due to forces caused by motion of the air in the balance case driven by thermal gradients. Two types of thermal problems may be distinguished. The first, which has received considerable recent study by Schoonover and Taylor [9], arises when the standard or the unknown is maintained at a different temperature from the balance chamber itself. If both standard and unknown are maintained at the same temperature offset from the balance temperature, errors may still be negligible provided the two objects have identical geometry.

A second thermal problem may arise from a heat source at some point around the balance case. The balance operator may be one such heat source. This effect is more difficult to study and has not received much recent attention in analytical weighing. Schürmann et al. produced a theory which applies to these types of forces and tested it with a series of experiments [10]. In one experiment, however, the observed force due to air convection was 40 times greater than the prediction. Some cancellation of unwanted effects should also be seen for this type of thermal problem if the standard and unknown have identical geometries.

## 4. Design of Calibration

Confronted with the need to calibrate a set of 590-g piston-gage weights to better than 1 ppm and faced with the set of known or suspected problems listed in the previous section, we designed the following measurement system. Weighings were carried out on a H315-MC Mettler balance^1^. This is a single-pan mechanical balance with I-kg capacity. We will refer to this balance as Bal-2. The balance was housed in a double-walled aluminum box with foam insulation between the walls. Standards and unknowns could both be stored inside the box. A small door in front of the box allows the operator to move objects manually on and off the balance pan. A small window allows the operator to observe the balance chamber and the scale reading. The balance arrestment mechanism is fed through the outer box and thus can be manipulated remotely by the operator.

Each piston-gage weight (X) is approximately washer-shaped. Pertinent physical properties are shown in table 1. The outer diameter of each weight is too large to permit it to lie flat on the balance pan. Instead, it is hung from a hook centered between the two vertical pan supports. The hook is at sufficient height so that the suspended weight clears the pan. Based on considerations in section 3, minimum errors should be obtained when weighing by double substitution [11] against a standard of similar geometry which is also suspended from the same hook. Thus one of the piston-gage weights (X1, say) should serve as the standard for calibrating the remaining weights. The problem then reduces to calibrating X1 the best way possible.

The best balance available to us was the NBS primary kilogram comparator (Bal-1). This operates at a fixed load of 1-kg and has a precision of 1.5 *μ*g (one standard deviation) for a single observation. Of equal importance, all manipulations are automated and carried out by remote control. Thus temperature gradients within the balance chamber do not exceed 5 mK side-to-side. A vertical temperature gradient of about +0.5 mK/cm is imposed for stability [12].

There is, unfortunately, no way in which X1 can be calibrated directly on this balance. Instead, we constructed a transfer standard (T) whose physical properties are also given in table 1. The virtue of T is that it can be calibrated using the kilogram comparator and then used to calibrate X1. When being calibrated, it lies flat with an additional 410 g of calibrated standards placed on top. When used to calibrate X1, T can be suspended from the hook. A hole 1 cm in diameter is drilled through T for this purpose. The placement of the hole was determined by constraining the center-of-mass of T to have the same elevation as that of X1 when suspended from the same hook. When compared on Bal-2, the two weights T and X1 are stored outside the balance (but within the insulated box) hanging side-by-side on hooks at the same elevation as the hook within the balance. The weights were always allowed to equilibrate overnight before measurements were begun.

As a check on the measurement scheme, we also calibrated X1 and T directly against the same set of standards using Bal-2. Both X1 and T were hung from the hook and stored outside the balance as described above. The collection of standard weights was stored inside the box on an aluminum plate at the same elevation as the balance pan. While we might expect systematic errors from various sources in each calibration of X1 and T, we do not expect that the difference in mass between X1 and T measured this way should differ significantly from that found from direct intercomparison. The effect of the gravitational gradient on these measurements requires a correction of about 0.015 mg, which was applied in order to obtain the results presented in this paper. As a matter of interest, we repeated the measurement of T against the set of standards. This time, however, T was placed flat on the balance pan and stored flat on the aluminum plate alongside the standards.

We also measured T and X1 against the same set of standards on a different balance. The balance was another H315-MC located in the NBS mass calibration laboratory (Bal-3). For this balance, the method described by Schoonover and Taylor for minimizing thermal problems is used [8]. In this scheme, there is no box around the balance. Instead, a massive aluminum plate is placed next to the balance. Active servo-control maintains the plate at the same temperature as a probe located at the front of the weighing chamber. Weights placed on the plate are covered with an aluminum can and allowed to equilibrate overnight. The thermal load represented by the operator is simulated by a heating element when the operator is not present. Using this set-up, T and X1 were placed flat on the plate in order to come into thermal equilibrium. During balance observations, T was placed flat on the pan and X1 was tilted so that it was constrained at top and bottom by the two pan supports (i.e., this balance was not equipped with a hook for weighing X1).

## 5. Results

### 5.1 Calibration of T (Bal-1)

As mentioned above, T was calibrated using the NBS primary kilogram comparator. We did not have an opportunity to search for a possible between-times component to this calibration. However, such a component is negligible in the comparison of platinum/10 percent iridium and stainless-steel kilograms, which differ markedly in physical properties. The result of the calibration of T is shown in table 2 along with the estimated standard deviation. All known errors reported in this and succeeding tables have been estimated by statistical methods. Errors estimated in this way are sometimes referred to as Type A errors.

### 5.2 Calibration of X1 (Bal-2)

The two weights T and X1 were compared on four different days. The result of each day’s comparison was taken as the average of at least four duplicate measurements. No between-times component is discernible within the limited sample of measurements. The standard deviation *σ*_w_ of the balance used is taken to be 40 *μ*g. This number is based on a large number of degrees of freedom. Each day’s results are considered to be representative of the long-term standard deviation so long as they pass an F-test at the 90 percent level of confidence. The mass value assigned to X1 is based on the measured difference between T and X1 and the assigned mass value of T. Results are summarized in table 3.

### 5.3 Check of Closure (Bal-2)

A set of calibrated standards was used to calibrate X1 and T in separate experiments. Again, no between-times component to the uncertainty was observed. The results are summarized in table 4.

### 5.4 Check Using Bal-3

The same standards used in the closure check were also used to calibrate T and X1 on Bal-3. In this case, T was measured when placed flat on the pan and X1 was measured as described above in section 5. The results are shown in table 5.

## 6. Discussion

There are several striking discrepancies in the results at the level of about 0.3 ppm in the mass of X1 or T. Perhaps the most interesting of these is that, when the same standard weights were used with Bal-2, the mass value computed for T depended on whether T was suspended from the hook or was placed flat on the pan. As seen in table 4, the difference in these two results is 188 *μ*g, more than seven times greater than the combined standard deviation based on the random errors of each measurement. The discrepancy cannot be explained by changes in the ambient relative humidity. We reoriented the hook and repeated the measurements to check whether geometrical imperfections in the balance could be the cause of the discrepancy. The results, however, were the same. The mass obtained when T was flat is consistent with that obtained on Bal-1. The latter measurement is assumed free from significant errors due to lack of thermal equilibrium.

The difference in mass between T and X1 when measured directly (mass of X1 from table 3 subtracted from mass of T from table 2) and when inferred from measurements of each weight against a common set of standards (mass of X1 subtracted from mass of T, both from table 4 in hanging orientation) disagree by 50 *μ*g. This represents 2.8 times the combined standard deviation assigned to the results. Again, differences in the ambient relative humidity cannot reasonably explain the discrepancy. As an indication of our inability to offer any plausible explanation for this lack of agreement, we will include an additional 50 *μ*g error to the mass value assigned to X1, giving a new total of 0.057 mg (0.1 ppm).

The results obtained on Bal-3 are also interesting. In this case, the value found for the mass of T agrees rather well (1.1 times the combined standard deviations) with the accepted value obtained on Bal-1. The result for X1, however, is significantly lower than the value shown in table 3. It is, perhaps, noteworthy that the discrepancies found on Bal-3 are anticorrelated with those obtained from similar measurements on Bal-2. In fact the average of the discrepant measurements taken on Bal-2 and Bal-3 (that is, direct calibrations of T and X1 against the same set of standards) agrees well with the values shown in tables 2 and 3. In retrospect, it would have been interesting to attempt these measurements on Bal-2 or Bal-3 with no auxiliary apparatus to bring the weights into good thermal equilibrium with the balance chamber—this is the condition in most laboratories and, we suspect, might lead to even larger errors than those we encountered.

Finally, we wish to emphasize that the observed discrepancies cannot be explained by errors in the correction due to air buoyancy [eq (1)], as a calculation will now show. If the air density within the weighing chamber has a significant vertical gradient, then eq (1) must be modified:

$$m_{X}=m_{S}\cdot (1-\rho_{L}/\rho_{S})/(1-\rho_{U}/\rho_{X})+\Delta .$$ (1a)

In eq (1a), *ρ*_L_ is the mean air density in the vicinity of the standards which are placed on the pan in the lower part of the balance chamber. Similarly, *ρ*_U_ is the mean air density in the vicinity of T when it is hanging from the hook near the top of the balance chamber. If one were mistakenly to assume that *ρ*_U_ *= ρ*_L_, then *m*_X_ would be overestimated by approximately

$$m_{S}(\rho_{L}-\rho_{U})/\rho_{X}.$$

In order to account for the observed discrepancies found when Bal-2 was used to measure T flat on the pan and suspended from the hook, there would need to be a difference in air density of about 0.2 percent from the pan to a point about 9 cm above the pan. This translates to a gradient in pressure of 22 Pa/cm or a temperature gradient of 70 mK/cm. Both these numbers are unrealistically large.

## 7. Conclusions

We believe that the mass assigned to X1 in table 3 has a total uncertainty of 0.057 mg and that this uncertainty approaches the best results which can be achieved by conventional. weighing techniques using a commercial analytical balance. There is some indication that, even when thermal problems of the type examined by Schoonover and Taylor have been overcome, other problems remain. Perhaps the origin of these additional problems can be found in the model of Schürmann et al. In addition, we have pointed to questions concerning changes in the mass of piston-gage weights between atmospheric and vacuum conditions due to loss of surface moisture. This uncertainty is of equal magnitude to the present uncertainty of the mass of these weights as determined from weighing in air. We have not studied questions of routine cleaning and handling of piston-gage weights, although such questions may be pertinent.

Improvement in the assignment of mass to piston-gage weights used in the absolute mode would require two innovations. First, the weights themselves must be reduced in surface area as much as possible. Since the shape of the weights cannot practically be changed, the only improvement possible is to polish the weights to a specular finish. Second, the mass of weights should, ideally, be determined in vacuo. Since mass standards are themselves calibrated in air, use of mass standards in vacuo to calibrate a piston-gage weight would require additional surface studies. Vacuum weighing, on the other hand, offers the simplification that buoyancy corrections and air convection are negligible. This advantage may prove to be overwhelming if improved accuracy is required.

## Figures and Tables

**Table 1:** Pertinent physical characteristics of the weights designated X and T in the text

	X	T
Nominal mass (g)	590	590
Density at 20°C (kg·m^−3^)	7837	7868
O.D. (mm)	135	75
I.D. (mm)	70	n.a.
Thickness (mm)	7	17
Surface area (cm^2^)	250	130

**Table 2:** Mass value and error budget for calibration of T on Bal-1

Mass of T: 590.049 383 g	
Uncertainty:	
	Type A
Reference standards	0.024 mg
Buoyancy correction	nil
Standard deviation	0.002 mg
RSS total	0.024 mg

**Table 3:** Mass value and error budget for calibration of X1 on Bal-2

Mass of X1: 590.034 153 g	
Uncertainty:	
	Type A
Reference standard (T)	0.024 mg
Buoyancy correction	
from *ρ*_a_	nil
from *ρ*_X_	0.006 mg
Mean standard deviation	0.012 mg
RSS total	0.027 mg

**Table 4:** Mass values and error budget for calibration of X1 and T with respect to the same standards; measurements on Bal-2

(1) Mass of X1 (hanging):	590.034232 g
(2) Mass of T (hanging):	590.049 513 g
(3) Mass of T (flat):	590.049 325 g
Uncertainty:	
	Type A
Reference standards	0.022 mg
Buoyancy correction	nil
Mean standard deviation	0.004 mg (1)
	0.018 mg (2), (3)
RSS total	0.022 mg (1)
	0.028 mg (2), (3)

**Table 5:** Mass values and error budget for calibration of X1 and T with respect to the same standards; measurements on Bal-3

Mass of X1 (tilted):	590.033 986 g
Mass of T (flat):	590.049 424 g
Uncertainty:	
	Type A
Reference standards	0.022 mg
Buoyancy correction	nil
Mean standard deviation	0.016 mg
RSS total	0.027 mg (1)

## Footnotes

1 Brand names are used solely to identify the apparatus used. Such identification neither implies endorsement by the National Bureau of Standards, nor does it imply that the particular product or equipment is necessarily the best available for the purpose.
